# *Simaonukia*, a new genus of the leafhopper tribe Evacanthini (Hemiptera, Cicadellidae, Evacanthinae), with descriptions of a new species from China

**DOI:** 10.3897/zookeys.669.5952

**Published:** 2017-04-21

**Authors:** Yujian Li, Zizhong Li, Maofa Yang

**Affiliations:** 1 Institute of Entomology, Guizhou University, Guiyang, Guizhou Province 550025, China; 2 School of Life Science, Qufu Normal University, Qufu, Shandong Province 273165, China

**Keywords:** Homoptera, Auchenorrhyncha, morphology

## Abstract

Simaonukia, a new leafhopper genus of Evacanthini (Hemiptera, Cicadellidae, Evacanthinae), and a new species, Simaonukia longispinus **sp. n.** from Yunnan, China are described.

## Introduction

Evacanthini is a relatively small leafhopper tribe of the subfamily Evacanthinae (Cicadellidae) with most species (more than 220 species) present in China (see Li and Wang 1996). While sorting and identifying the evacanthine leafhopper material in Institute of Entomology of Guizhou University, we found a new genus and species which we describe here. The type specimen is deposited in the Institute of Entomology, Guizhou University, Guiyang, China (GUGC).

## Taxonomy

### Simaonukia

Taxon classificationAnimaliaHemipteraCicadellidae

Li & Li gen. n.

http://zoobank.org/F420F245-01D3-4474-A418-C6B52FFD9B5C

#### Type species.

*Simaonukia longispinus* sp. n.

#### Description.

**Figures 1–5.**
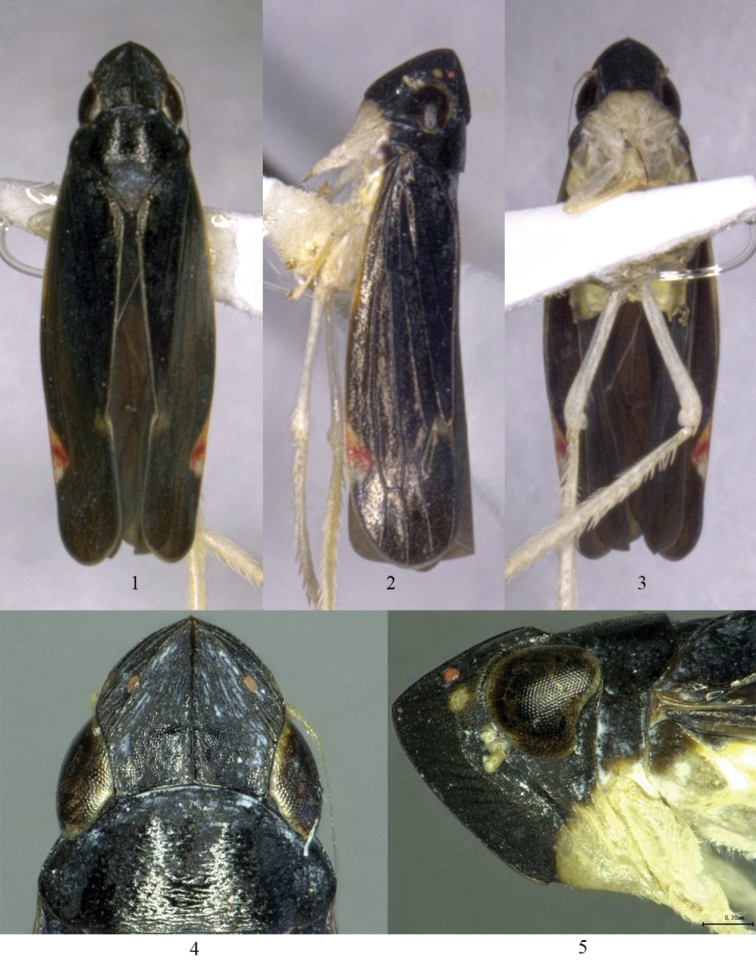
*Simaonukia longispinus* sp. n. **1–3** Male, dorsal view, lateral view and ventral view **4–5** Head, dorsal view, lateral view.

Body medium-sized, usually black. Head (Figs 1, 4) in dorsal view narrower than pronotum; with five distinct carinae, a median carinae, two lateral carinae and two subocellar carinae converging to apex of vertex, area between median carina and submarginal carina with many fine longitudinal wrinkles; disc with a short transverse ridge basally, carinate. Front of head (Figs 1, 4) slightly conically produced; vertex about as long as or a little longer than pronotum and nearly two times longer and three times wider than eye. Ocelli (Figs 1, 2, 4, 5) placed just laterad of lateral carina, well in front of eye. Face (Fig. 3) including eyes shorter than wide; frontoclypeus (Figs 2, 3, 5) tumid, with median longitudinal carina strongly elevated; clypellus broad and swollen at base; lora nearly reaching middle of clypellus. Pronotum (Figs 1, 4) with sides strongly convergent cephalad. Scutellum (Fig. 1) with transverse depression distinct. Hind femur (Fig. 11) with apical macrosetal formula 2+1+1. Forewing (Figs 1–3) with R_1a_ present; with four apical cells and two closed subapical cells; appendix very narrow.

Male genitalia. Pygofer without ventral process, triangular in lateral view, with some small setae on ventral side near apex. Subgenital plate ligulate with many macrosetae and long fine setae ventrally. Aedeagal shaft short with pair of lamellae dorsally near base and pair of lateral processes subbasally. Style short, foot-like apically. Connective similar in length to style, arms very short.

#### Distribution.

China (Yunnan).

#### Etymology.

The genus name is formed from a combination of the collection locality and the similar evacanthine genus *Onukia* Matsumura.

#### Remarks.

**Figures 6–15.**
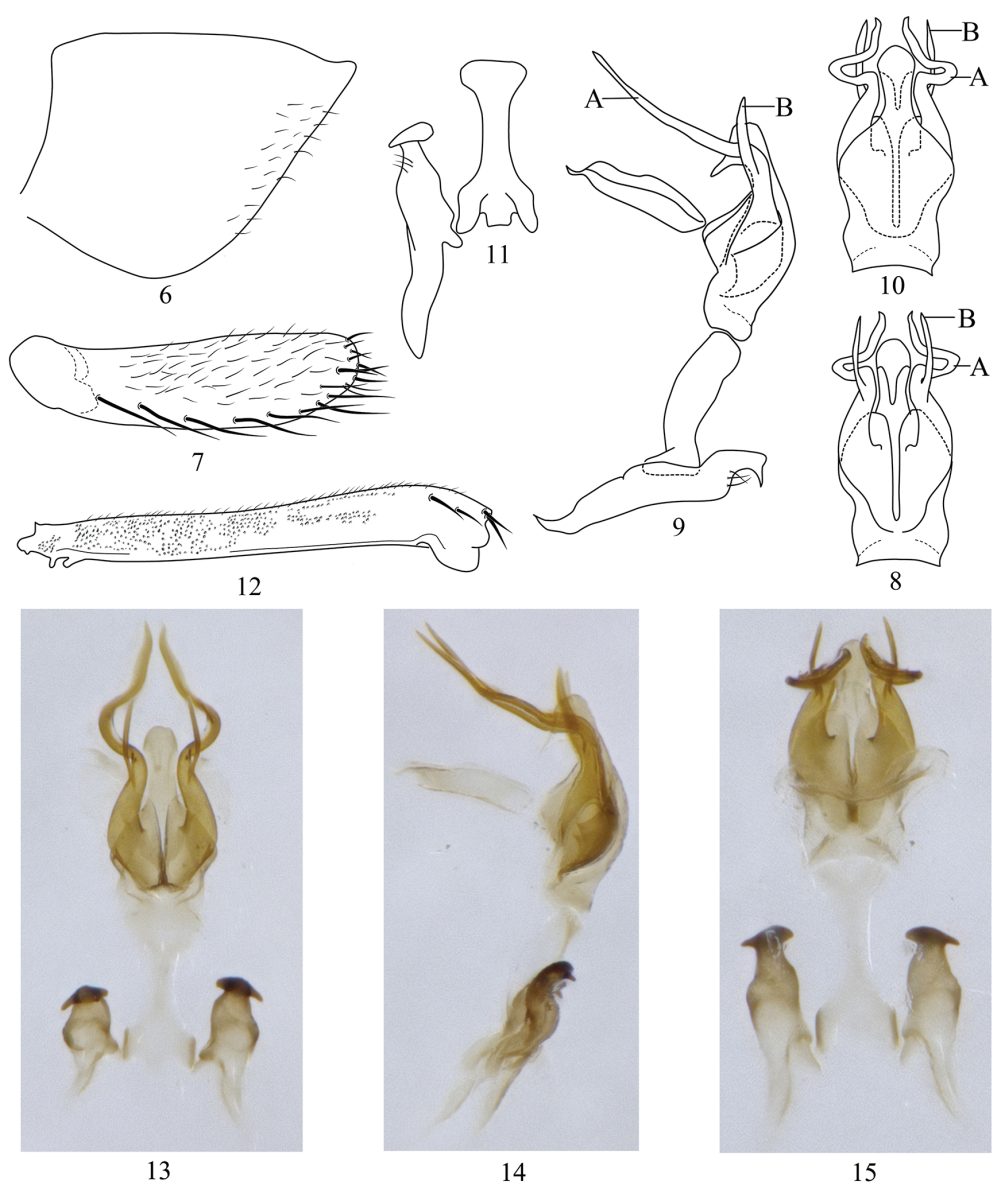
*Simaonukia longispinus* sp. n. **6** Male pygofer, lateral view **7** Subgenital plate **8** Aedeagus, ventral view **9** Aedeagus, style and connective, lateral view **10** Aedeagus, dorsal view **11** Style and connective **12** Hind femur **13** Aedeagus, style and connective, ventral view **14** Aedeagus, style and connective, lateral view **15** Aedeagus, style and connective, dorsal view.

This genus can be distinguished by the moderately conically produced head with five distinct longitudinal carinae and dense striations and a transverse carinate ridge basally on disk. In the key to genera by Wang et al. (2015) the new genus runs to *Onukia* Ishihara but can be distinguished by: 1) Aedeagus (Figs 8–10, 13–15) with a pair of lateral processes subbasally; 2) Pygofer (Fig. 6) without processes.

### Simaonukia longispinus

Taxon classificationAnimaliaHemipteraCicadellidae

Li & Li sp. n.

http://zoobank.org/8FF80210-F181-4DDD-B902-0A329C574FF5

Figs 1–5 , 6–15

#### Measurements.

body length (including forewing): ♂: 4.9 mm.

Vertex, pronotum and scutellum black (Figs 1, 2, 4, 5). Frontoclypeus black, anteclypeus light yellow (Figs 2, 3, 5). Forewing black, with nearly pale white and subtranslucent plaque in middle of costal area and around R_1a_, area along R_1a_ red (Figs 1–3).

Male pygofer (Fig. 6) without ventral process, with a hyaline lateral stripe near middle area. End of style foot-like (Figs 9, 11). Subgenital plate ligulate, blunt at base, with a uniseriate row of many macrosetae on ventral surface and many moderately long fine setae laterally (Fig. 7). Aedeagal shaft dorso-ventrally compressed, tapering to digitate apex, the latter with a dorsal flange-like acute process (Figs 9, 14); with a pair of very long lateral subbasal processes (A in Figs 8–10), directed posteriorly then sharply turned dorsally near midlength with apex sinuate, with short sub-basal process (B in Figs 8–10).

#### Type material.

Holotype: ♂, CHINA, **Yunnan**: Puer, Simao, Caiyanghe, 24 August 2014, coll. Guo Meina.

#### Etymology.

The species name refers to the long lateral spine of the aedeagus.

#### Remarks.

This species can be distinguished by the dorsum and upper part of face blackish brown and area along R_1a_ in the forewing red and in the male genitalia by the elongate lateral processes of the aedeagus bifurcate sub-basally.

## Supplementary Material

XML Treatment for Simaonukia

XML Treatment for Simaonukia longispinus
